# Anemia and its associated factors among women of reproductive age in eastern Africa: A multilevel mixed-effects generalized linear model

**DOI:** 10.1371/journal.pone.0238957

**Published:** 2020-09-11

**Authors:** Achamyeleh Birhanu Teshale, Getayeneh Antehunegn Tesema, Misganaw Gebrie Worku, Yigizie Yeshaw, Zemenu Tadesse Tessema

**Affiliations:** 1 Department of Epidemiology and Biostatistics, Institute of Public Health, College of Medicine and Health Sciences, University of Gondar, Gondar, Ethiopia; 2 Department of Human Anatomy, College of Medicine and Health Science, School of Medicine, University of Gondar, Gondar, Ethiopia; 3 Department of Physiology, School of Medicine, College of Medicine and Health Sciences, University of Gondar, Gondar, Ethiopia; University of Mississippi Medical Center, UNITED STATES

## Abstract

**Background:**

Anemia in women of reproductive age is a major public health challenge for low- and middle-income countries with a long-term negative impact on the health of women, their children, and the economic growth of the society. Even though the world health organization targeted a 50% global reduction of anemia among women of reproductive age by 2025, with the current trend it is unlikely to achieve this goal.

**Objective:**

This study aimed to assess the prevalence and associated factors of anemia among women of reproductive age in eastern Africa.

**Methods:**

A secondary data analysis, using demographic and health survey (DHS) data of 10 eastern African countries, was conducted. For our study, a total weighted sample of 101524 women of reproductive age was used. We employed a multilevel mixed-effects generalized linear model (using Poisson regression with robust error variance). Both unadjusted and adjusted prevalence ratios with their 95% confidence interval were reported.

**Results:**

The prevalence of anemia in eastern Africa was 34.85 (95%CI: 34.56–35.14) ranging from 19.23% in Rwanda to 53.98% in Mozambique. In the multivariable multilevel analysis, being older age, having primary and above education, being from households with second to highest wealth quantiles, being currently working, not perceiving distance as a big problem, use of modern contraceptive methods, and rural residence was associated with a lower prevalence of anemia. While, being married and divorced/separated/widowed, women from female-headed households, women from households with unimproved toilet facility and unimproved water source, ever had of a terminated pregnancy, having high parity, and being from large household size was associated with a higher prevalence of anemia.

**Conclusion:**

The prevalence of anemia in eastern Africa was relatively high. Both individual level and community level factors were associated with the prevalence of anemia in women of reproductive age. Therefore, giving special attention to those women who are at a higher prevalence of anemia such as younger women, those who are from households with low socioeconomic status, unimproved toilet facility, and source of drinking water, as well as pregnant women could decrease anemia in women of reproductive age.

## Background

Anemia is a condition in which the number of healthy red blood cells/ hemoglobin (Hgb) level (and consequently their oxygen-carrying capacity) is insufficient to meet the body’s physiologic needs [1, 2]. Anemia affects more than 500 million women of reproductive age globally and it is a major public health challenge for low- and middle-income countries (LMICs) with a long-term negative effect on the health of women, their children, and the economic growth [3–5].

Anemia in women of reproductive age has a tremendous effect on the women such as; loss of productivity due to reduced work capacity, cognitive impairment, increased susceptibility to infections due to its effect in immunity, stillbirth/miscarriage, and maternal mortality [6–10]. Besides, anemia in women of reproductive age can result in poor feto-neonatal outcomes such as preterm birth, low birth weight, depletion of the iron stores of the newborn, and in general, it may end up with infant/child mortality [9–13].

The most common type of anemia worldwide is nutritional anemia mainly due to iron, folate, and vitamin B12 deficiencies. Iron deficiency anemia is the most common cause of anemia, with over 50% of anemia are due to iron deficiency [14–16]. Iron deficiency is common in women of reproductive age because of their high demand for iron during pregnancy, lactation, menstrual blood loss, and nutritional deficiencies during their reproductive cycle [9, 17].

Globally, in 2011, the prevalence of anemia in pregnant women was 38% and in non-pregnant women was 29% [18]. Even though anemia affects all countries, it mostly affects LMICs especially Asian and Sub-Saharan African countries which accounts for 89% of the anemia burden [19]. In eastern Africa, the prevalence of anemia in women of reproductive age is higher, which ranges from 19.2% in Rwanda to 49% in Zambia [20–26].

According to different studies done worldwide; age [27, 28], educational level [29–31], occupation [32, 33], marital status [20, 34, 35], wealth status [20, 21, 29, 30, 36], sex of household head [32, 37, 38], media exposure [39–41], body mass index [20, 29, 35, 42], type of toilet facility and source of drinking water [21, 29], ever had of terminated pregnancy [39, 43, 44], parity [36, 45], household size [46, 47], modern contraceptive use [20, 48], current pregnancy status [21, 28, 30, 35, 45], currently breastfeeding [30, 39], residence [49], and community literacy level [32] are associated with anemia in women of reproductive age.

The world health organization (WHO) puts anemia as a public health problem if it is greater than 5% [50], but most of the studies indicated above revealed that the prevalence of anemia in women of reproductive age is above 20%. Also, WHO has set a global target of achieving a 50% reduction of anemia in women of reproductive age by 2025, even though it is unlikely to achieve this plan with the current trend [51]. Therefore, this study aimed to assess the prevalence and associated factors of anemia in women of reproductive age. We hypothesized that the prevalence of anemia in women of reproductive age in eastern Africa is high and different factors are associated with anemia development. The findings of this study will have an advantage in informing policymakers and program planners for making better decisions and plan appropriate intervention strategies to tackle this major public health problem and achieve the plan set by the WHO.

## Methods

### Data source, sampling technique, and population

This study was based on the current 10 Demographic and Health Surveys (DHS) conducted between 2008 and 2018 in Eastern African countries; Burundi, Ethiopia, Malawi, Mozambique, Rwanda, Tanzania, Uganda, Zimbabwe, Madagascar, and Zambia, since the rest two east African countries (Kenya and Comoros) had no recorded anemia or hemoglobin level in the data set, after appending the data sets. The DHS used the stratified cluster sampling technique by using their respective population and housing census as a sampling frame [52]. For this study, we used a weighted sample of 101524 women of reproductive age.

### Variables of the study

#### Dependent variable

This study was based on altitude adjusted hemoglobin level, which was already provided in the DHS data. The outcome variable was anemia level, which was measured based on women's pregnancy status as; if pregnant a hemoglobin value <11 g/dL, and if non-pregnant a hemoglobin value <12 g/dL is considered anemic. In addition, based on severity anemia was classified as severe (if Hgb value <7 g/dL), and moderate (if Hgb value 7–9.9 g/dL) in women of reproductive age and mild (if Hgb level is 10.0–10.9 g/dL) in pregnant women and non-pregnant women (if Hgb level is 10.0–11.9 g/dL). For this study, we re-categorized anemia level as anemic coded as “1” and non-anemic coded as “0” from the previous classifications (no, mild, moderate, and severe) since there were very small numbers of cases in the categories of severe and moderate anemia.

#### Independent variables

After reviewing of literature, both individual and community level explanatory variables were considered. Individual level variables included were; age of the respondent, educational level, occupation, marital status, wealth status, sex of household head, media exposure (constructed from three variables; frequency of listening radio, frequency of watching television, and frequency of reading newspaper), type of toilet facility, source of drinking water, ever had of a terminated pregnancy, parity, household size, perception of distance from the health facility, modern contraceptive use, current pregnancy status, and breastfeeding. Residence, community literacy level, and community poverty level were included as community level variables. Community literacy level and poverty level were created by aggregating individual level variables at cluster/community level since these variables are found to be factors for anemia and not directly found in the DHS.

**Community poverty level**: is the proportion of women in the community who have low household wealth quantiles (lowest and second quantiles).

**Community literacy level**: is the proportion of women in the community who have primary and above educational levels.

To categorize as low and high we used national median value (<50 as low and ≥ as high) since these variables were not normally distributed.

### Data management and statistical analysis

Extraction, further coding, and both descriptive and analytical analysis were carried out using STATA version 14 software. Weighting was done throughout the analysis to take into account/adjust disproportional sampling and non-response as well as to restore the representativeness of the sample so that the total sample looks like the country’s actual population. Descriptive analysis was carried out using frequencies and percentages. The multilevel model was fitted due to the hierarchical nature of the DHS data. In our study, since the prevalence of anemia was high and the outcome was binary, we employed a multilevel mixed-effects generalized linear model (using Poisson regression with robust error variance). Besides, the Intra-class Correlation Coefficient (ICC), the Proportional Change in Variance (PCV), and the Median odds Ratio (MOR) were reported to check whether there was a clustering effect/variability. Bi variable analysis was first done to select variables for multivariable analysis and variables with p-value <0.20 in the bivariable analysis were eligible for the multivariable analysis. While doing the multilevel analysis four models; the null model (containing outcome variable only), model I (containing only individual level variables), model II (containing community level variables only), and model III (incorporating both individual and community level variables simultaneously) were fitted. Model comparison was done using deviance and unadjusted and adjusted prevalence ratio (PR) with 95% confidence interval (CI) was reported for the best-fitted model. Finally, variables with p-value <0.05 in the multivariable multilevel regression analysis were considered to be significant factors associated with the prevalence of anemia in women of reproductive age.

### Ethical consideration

Since this is a secondary analysis of DHS data, ethical approval was not necessary. But we registered and requested access to these DHS datasets from DHS on-line archive and received approval to access and download the data files.

## Results

### Sociodemographic characteristics

For this study, we used a total weighted sample of 101524 women of reproductive age with the majority (14.70%) of the participants from Ethiopia. The median age of the study participants was 28 (IQR = 20–35) years with the majority (21.97%) between 15 to 19 years. Most (47.86%) of our study participants had primary education and 62.18% of them had got married. Around one fourth (24.23%) of participants were from households with the highest wealth quintile and 56.75% of respondents had a job/ currently working. Regarding sex of household head and media exposure, about 70.45% and 66.79% of respondents were from male-headed households and had media exposure respectively. More than two-thirds (69.85%) of participants were from households with an improved water source and only 44.71% of participants were from households with improved toilet facility. Regarding parity, 34.68% and 26.90% of respondents were multiparous and nulliparous respectively. Most (59.02%) of respondents did not perceive distance from the health facility as a big problem and around three fourth (71.56%) of respondents were rural dwellers (Table 1).

**Table 1:** Sociodemographic characteristics of respondents.

Variables	Frequency	Percentage
Country		
Burundi	8587	8.46
Ethiopia	14923	14.70
Madagascar	8308	8.18
Malawi	7933	7.81
Mozambique	13571	13.37
Rwanda	6680	6.58
Tanzania	13063	12.87
Uganda	5988	5.90
Zambia	13234	13.04
Zimbabwe	9236	9.10
Age (years)		
15–19	22301	21.97
20–24	18900	18.62
25–29	17163	16.91
30–34	14632	14.41
35–39	12165	11.98
40–44	9286	9.15
45–49	7077	6.97
Educational level		
No education	21503	21.18
Primary	48585	47.86
Secondary	27817	27.40
Higher	3619	3.56
Marital status		
Never married	26233	25.84
Married	63127	62.18
Divorced/widowed/separated	12164	11.98
Occupation		
Working	57612	56.75
Not working	43912	43.25
Household wealth quintile		
Lowest	18306	18.03
Second	18651	18.37
Middle	18940	18.66
Fourth	21025	20.71
Highest	24602	24.23
Sex of household head		
Male	71520	70.45
Female	30004	29.55
Media exposure		
Yes	67811	66.79
No	33713	33.21
Type of toilet facility		
Improved	45387	44.71
Unimproved	56137	55.29
Source of drinking water		
Improved	70913	69.85
Unimproved	30611	30.15
Ever had of a terminated pregnancy		
Yes	11622	11.45
No	89902	88.55
Parity		
None	27310	26.90
Primiparous	14851	14.63
Multiparous	35205	34.68
Grand multiparous	24158	23.80
Household size		
1–2	7899	7.78
3–5	44659	43.99
6 and above	48966	48.23
Distance from the health facility		
Big problem	41604	40.98
Not a big problem	59920	59.02
Modern contraceptive use		
Yes	27905	27.49
No	73619	72.51
Currently pregnant		
Yes	8511	8.38
No/unsure	93013	91.62
Currently breastfeeding		
Yes	27690	27.27
No	73834	72.73
Residence		
Urban	28871	28.44
Rural	72653	71.56
Community poverty level		
Low	50845	50.08
High	50679	49.92
Community literacy level		
Low	52180	51.40
High	49344	48.60

### Prevalence of anemia among women of reproductive age in eastern Africa

**Fig 1:**
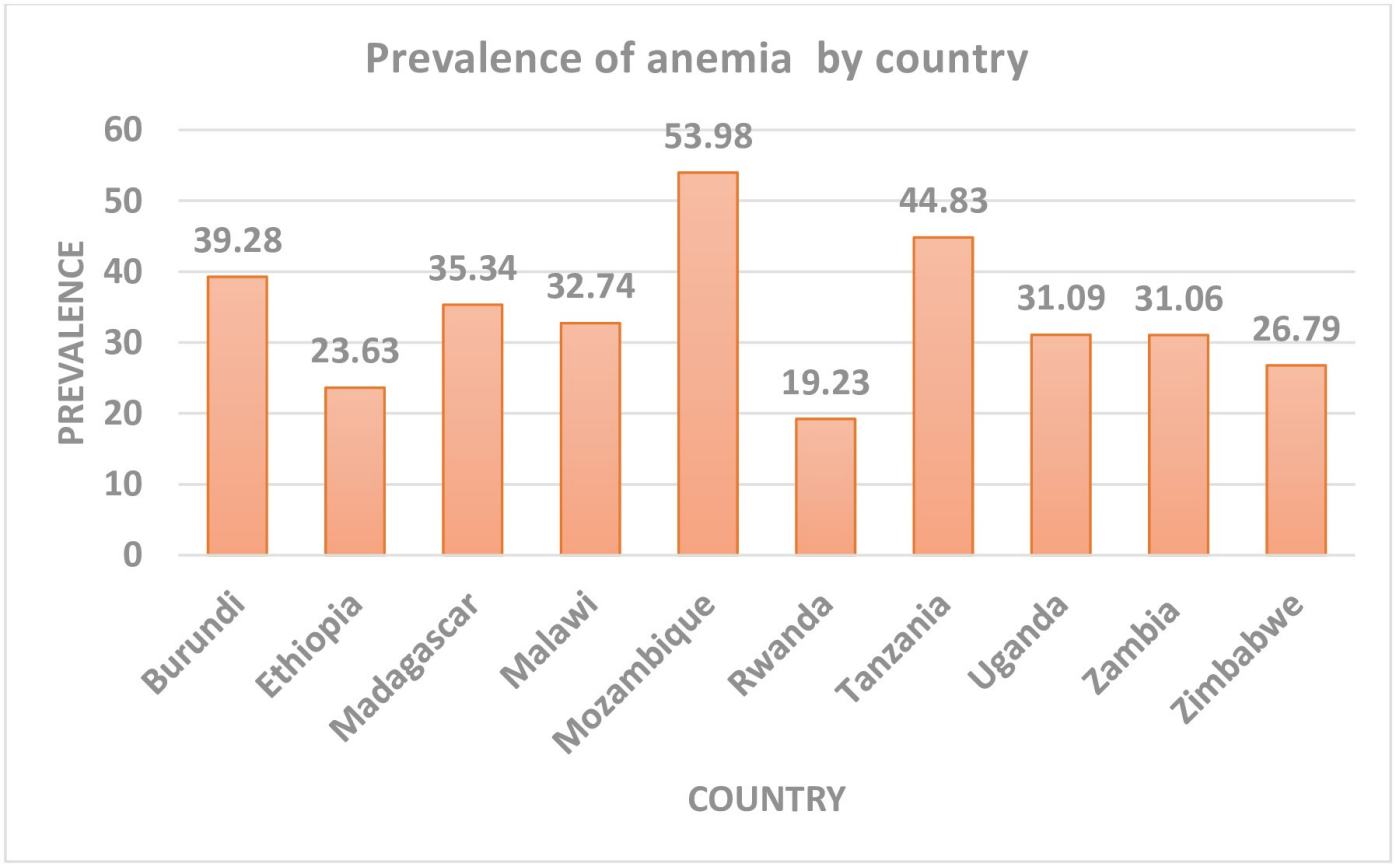
Prevalence of anemia in eastern Africa showing great variation between countries.

**Fig 2:**
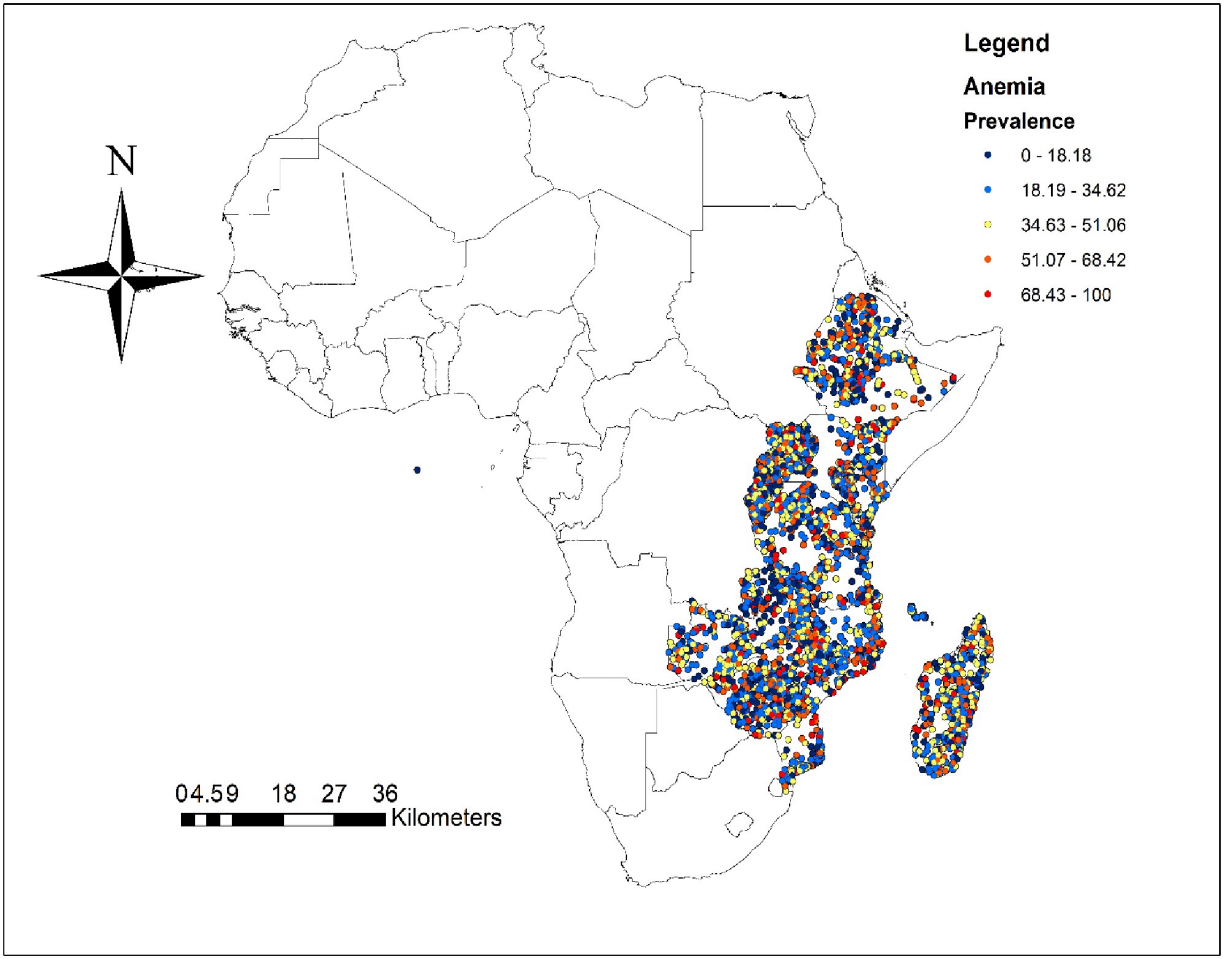
Spatial distribution of anemia among women of reproductive age in eastern Africa.

The prevalence of anemia in reproductive age women in eastern Africa was 34.85 (95%CI: 34.56–35.14) with huge variation between countries ranged from 19.23% in Rwanda to 53.98% in Mozambique (Fig 1). Fig 2 shows the spatial distribution of anemia in eastern Africa with the red dots indicating areas with the highest prevalence of anemia.

### Random effects analysis/variability

The community level variability was assessed by both ICC and MOR. As shown in Table 2 the ICC and the MOR values in the null model, which was 6% and 1.54 respectively supports that there was clustering or community level variability of anemia. In addition, the highest PCV in the final model (model 3) revealed that higher proportions of the variation of anemia in women of reproductive age were explained by both individual level and community level factors. Regarding model comparison, deviance was used to select the best fit model among the four models. The model with the lowest deviance, the final model (model III) which incorporates both individual and community level factors simultaneously, was selected as the best-fitted model and we used it to assess the factors associated with anemia among women of reproductive age in eastern Africa (Table 2).

**Table 2:** Community level variability and model fitness for assessment of anemia among women of reproductive age in eastern Africa.

Parameter	Null model	Model I	Model II	Model III
Community level variance	0.21	0.20	0.16	0.15
ICC	0.06	0.05	0.06	0.04
MOR	1.54(1.48–1.58)	1.53(1.47–1.58)	1.46(1.41–1.52)	1.44(1.39–1.51)
PCV (%)	Reference	5%	24%	29%
**Model fitness**				
Deviance (-2LL)	145704	144310	145622	144228

### Factors associated with anemia among women of reproductive age in eastern Africa

All variables (both individual level and community level variables) had p-value <0.20 in the bivariable analysis and were eligible for multivariable analysis. In the multivariable analysis; the individual level factors such as age, education, marital status, occupation, household wealth status, sex of household head, type of toilet facility, source of drinking water, ever had of a terminated pregnancy, parity, household size, perception of distance from the health facility, and pregnancy status were significant determinants of anemia among women of reproductive age. Among community level factors, residence was significantly associated with anemia in women of reproductive age (Table 3).

**Table 3:** Bi variable and multivariable multilevel regression analysis to assess factors associated with anemia among women of reproductive age in eastern Africa.

Variables	Anemia		Prevalence ratio(PR)	
	No	yes	uPR (95%CI)	aPR (95%CI)
Age (years)				
15–19	14467	7834	1.00	1.00
20–24	12365	6535	0.98(0.94–1.03)	0.96(0.93–0.99) *
25–29	11472	5610	0.96(0.93–0.99)	0.92(0.89–0.96) *
30–34	9692	4910	0.96(0.94–0.99)	0.91(0.88–0.95) *
35–39	7779	4386	1.04(1.01–1.07)	0.97(0.93–0.99) *
40–44	5890	3396	1.04(1.01–1.08)	0.96(0.91–0.99) *
45–49	4477	2600	1.03(0.99–1.07)	0.92(0.87–0.97) *
Educational level				
No education	12921	8582	1.00	1.00
Primary	31371	17214	0.87(0.4–0.89)	0.92(0.90–0.95) *
Secondary	19190	8627	0.77(0.75–0.79)	0.87(0.84–0.90) *
Higher	2660	959	0.66(0.62–0.72)	0.79(0.74–0.83) *
Marital status				
Never married	17673	8560	1.00	1.00
Married	41081	22046	1.07(1.05–1.10)	1.09(1.05–1.12) *
Divorced/widowed/separated	7388	4776	1.18(1.15–1.22)	1.15(1.11–1.19) *
Occupation				
Working	28616	15296	0.96(0.94–0.98)	0.97(0.95–0.99) *
Not working	37526	20086	1.00	1.00
Household wealth quintile				
lowest	10864	7442	1.00	1.00
second	11928	6723	0.90(0.87–0.93)	0.94(0.91–0.97) *
Middle	12376	6564	0.87(0.84–0.90)	0.93(0.90–0.96) *
Fourth	14090	6935	0.85(0.82–0.88)	0.93(0.90–0.97) *
Highest	16884	7718	0.78(0.75–0.81)	0.89(0.85–0.93) *
Sex of household head				
Male	47008	24512	1.00	1.00
Female	19134	10870	1.04(1.02–1.06)	1.05(1.02–1.07) *
Media exposure				
Yes	44647	23164	0.91(0.89–0.94)	1.02(1.01–1.04)
No	21495	12218	1.00	1.00
Type of toilet facility				
Improved	30689	14698	1.00	1.00
Unimproved	35453	20684	1.16(1.13–1.19)	1.05(1.02–1.07) *
Source of drinking water				
Improved	47338	23573	1.00	1.00
Unimproved	18804	11807	1.15(1.12–1.18)	1.04(1.01–1.07) *
Ever had of a terminated pregnancy				
Yes	7365	4257	1.07(1.04–1.09)	1.06(1.03–1.09) *
No	58777	31125	1.00	1.00
Parity				
None	18252	9057	1.00	1.00
Primiparous	9435	5416	1.07(1.05–1.11)	1.11(1.07–1.15) *
Multiparous	23216	11989	1.03(1.01–1.05)	1.07(1.03–1.12) *
Grand multiparous	15239	8919	1.11(1.09–1.14)	1.06(1.01–1.11) *
Household size				
1–2	5075	2824	1.00	1.00
3–5	29609	15050	0.95(0.91–0.98)	0.98(0.94–1.01)
6 and above	31458	17588	1.01(0.98–1.05)	1.05(1.01–1.09) *
Distance from the health facility				
Big problem	26239	15365	1.00	1.00
Not a big problem	39903	20017	0.90(0.88–0.92)	0.96(0.94–0.98) *
Modern contraceptive use				
Yes	20584	7321	0.70(0.68–0.71)	0.71(0.69–0.73) *
No	45558	28061	1.00	1.00
Currently pregnant				
Yes	4922	3590	1.24(1.20–1.27)	1.11(1.08–1.13) *
No/unsure	61221	31792	1.00	1.00
Currently breastfeeding				
Yes	17613	10077	1.06(1.04–1.08)	1.02(0.99–1.04)
No	48529	25305	1.00	1.00
Residence				
Urban	19069	9802	1.00	1.00
Rural	47073	25580	1.10(1.07–1.14)	0.94(0.90–0.98) *
Community poverty level				
Low	33499	17346	1.00	1.00
High	32643	18036	1.03(0.99–1.05)	0.97(0.95–1.01)
Community literacy level				
Low	33626	18554	1.00	1.00
High	32516	16828	0.94(0.92–0.97)	0.98(0.95–1.01)

aPR = adjusted Prevalence Ratio, uPR = unadjusted Prevalence Ratio,

* = p value<0.05.

Being in the older age group was associated with a lower prevalence of anemia as compared to the age group 15–19 years. The prevalence of anemia was 8%, 13%, and 21% lower in women had primary, secondary, and higher education, respectively, as compared with women with no formal education. The prevalence of anemia was 9% and 15% higher in women who were married and divorced/separated/widowed, respectively, as compared with those who were never married. The prevalence of anemia in currently working women was 3% lower as compared with their counterparts. Regarding household wealth quantiles, being women from second, middle, fourth, and highest household wealth quantiles was associated with 6%, 7%, 7%, and 11% lower prevalence of anemia as compared to those who were from the lowest household wealth quintile. Being women from female-headed households was associated with 5% higher prevalence of anemia as compared with male-headed households. Women from households with unimproved toilet facility and unimproved sources of water had 5% and 4% higher prevalence of anemia, respectively than their counterparts. Women with ever had of a terminated pregnancy had 6% higher prevalence of anemia as compared with their counterparts. Regarding parity of the respondent, primiparous, multiparous, and grand multiparous women had 11%, 7%, and 6% higher prevalence of anemia respectively, as compared to nulliparous women. Being women from larger household size (six and above) was associated with 5% higher prevalence of anemia as compared to those from households with a household size of one to two. Being a woman not perceiving distance from the health facility as a big problem was associated with 4% lower prevalence of anemia as compared to their counterparts. Using modern contraceptive methods was associated with 29% lower prevalence of anemia than women who were not using modern contraceptive methods. Being currently pregnant was associated with 11% higher prevalence of anemia as compared to non-pregnant women. Moreover, being from a rural area was associated with 6% lower prevalence of anemia as compared to urban areas (Table 3).

## Discussion

Anemia is a major public health problem in reproductive age women because of their high demand for iron during pregnancy, lactation, menstrual bleeding, and nutritional deficiency during their reproductive cycle [9]. This study assessed the prevalence of anemia and its associated factors among women of reproductive age in eastern African countries. In this study, the prevalence of anemia among women of reproductive age was 34.85 (95%CI: 34.56–35.14) and this is consistent with studies done in India and Nepal [53, 54]. Anemia prevalence in this study was higher than studies done in Brazil [27], Iran [36], Thailand [55], Turk [56], and Timor-Lest [31]. But this prevalence of anemia is lower than studies done in Nepal [34], Myanmar [35], Democratic Republic of Congo [28], India [57], and Vietnam [58]. This difference in anemia prevalence between countries may be due to the variation in geographical, cultural, and dietary-related factors between countries. In addition, the high prevalence of anemia among women in the countries of eastern Africa may be attributable to their social and biological susceptibility to anemia. Moreover, in developing countries especially in Eastern African countries, access to iron-rich food is inadequate due to their poor socioeconomic status, inadequate health care accesses, and utilization and this may result in anemia. In addition, this regional variation of anemia might be associated with the variation in the distribution and prevalence of communicable disease that commonly affect developing countries like eastern African countries.

Consistent with studies conducted elsewhere [21, 28–39, 45–49, 59], in our study, being older age, having primary and above education, being from households with second to highest wealth quintiles, being currently working, not perceiving distance as not a big problem, use of modern contraceptive methods, and rural residence was associated with a lower prevalence of anemia. While, being married and divorced/separated/widowed, women from female-headed households, women from households with unimproved toilet facility and unimproved water source, ever had of a terminated pregnancy, having high parity, and from large household size was associated with a higher prevalence of anemia.

In the study, the prevalence of anemia was lower among women who had primary and above education compared with those women who had no formal education. This finding is congruent with studies done in Ethiopia [30], Rwanda [29], Timor-Leste [31]. This might be because educated mothers usually eat a variety of foods such as vitamins and minerals which might lead to a reduction in nutritional deficiency anemia. In addition, obtaining education may help women adopt appropriate lifestyle patterns such as better health-seeking habits as well as hygiene practices that can prevent women from getting anemia. Consistent with other studies conducted in different settings [20, 21, 29, 30, 36, 60], in this study, being from second to highest household wealth quantiles were associated with lower prevalence of anemia as compared with women from households with lowest quantile. This could be due to improved socioeconomic status is associated with healthy nutrition, lower infection/morbidity, and increased access and utilization of medical health services [59, 61, 62]. In addition, it might be because of women from high socioeconomic status could purchase variety (both in quality and quantity) of foods.

The study at hand also revealed that being from households with unimproved toilet facility and unimproved sources of drinking water associated with a higher prevalence of anemia and this is in line with studies conducted Uganda and Ruanda [21, 29, 60]. This might be because women with unimproved toilet facility and unimproved sources of drinking water are at risk of both waterborne and foodborne diseases which might in turn, increases the risk of anemia. Moreover, these groups of women are at risk of getting helminthic infections such as hookworm, which is the most common cause of anemia in poor sanitary conditions. We also found that being pregnant was associated with a higher prevalence of anemia as compared with those who were not pregnant. This is in concordance with studies done in Jourdan [45], Democratic Republic of Congo [28], Rwanda [35], Ethiopia [30], and Uganda [21, 60]. This is due to the fact that pregnant women have an increased demand for iron to sustain her baby's development. Another possible explanation will be during pregnancy nutritional deficiencies, bacterial and parasitic infections, and genetic disorders of the red blood cells such as thalassemia is common, which could eventually lead to anemia [63]. Our study also revealed that modern contraceptive use was associated with anemia in women of reproductive age. Using modern contraceptive methods reduces the prevalence of anemia and this is in concordance with different studies [20, 32, 48]. This is because women who used modern contraceptive methods prevent complications related to pregnancy and childbirth, which could eventually reduce the prevalence of anemia due to recurrent blood loss. Another plausible explanation will be using modern contraception methods (especially hormonal contraceptive methods) could minimize the menstrual bleeding and reduce their susceptibility to anemia [64, 65].

Our study also found distance from the health facility as a significant anemia-related factor in which women who consider distance from the health facility as a major problem were at higher risk of anemia. This might be due to the fact that women who were far from the nearest facility cannot access maternal health services timely such as iron and folate supplementation during pregnancy, modern contraceptives as well as other services related to the continuum of care, which all make them susceptive to anemia. Moreover, in this study women from rural areas had lower odds of anemia as compared with those who were from urban areas. This is consistent with studies done in Malawi [49]. It might be because women living in rural areas usually have an increased access and utilization of Teff (a type of crop used to make Enjera, a traditional food in Ethiopia) and other iron-containing foods that can lead to a reduction in the risk of nutritional anemia.

This study was based on a multicounty analysis with a large sample size and appropriate statistical analysis considering the hierarchical nature of the DHS data. Therefore, we authors strongly believe that it provides more precise and generalizable findings that can be used by policymakers and program planners to design intervention strategies for the problem at both individual and community levels. However, this study was not without limitations. Due to the cross-sectional nature of the DHS data, we are unable to establish a cause and effect relationship between independent variables and anemia. Moreover, since the study was based on information available on the surveys, other confounders such as infections (such as malaria, intestinal parasites, and HIV/AIDS) were not adjusted.

## Conclusion

The prevalence of anemia in eastern Africa was relatively high. Both individual level and community level factors were associated with the development of anemia in women of reproductive age. Giving special attention for those groups of women who had a higher prevalence of anemia such as younger women, uneducated women, those who are from households with low socioeconomic status, unimproved toilet facility and source of drinking water is recommended.

## Abbreviations

aPR: Adjusted Prevalence Ratio
CI: Confidence Interval
DHS: Demographic and Health Surveys
ICC: Intra-class Correlation Coefficient
MOR: Median Odd Ratio
PCV: Proportional Change in Variance
uPR: unadjusted Prevalence Ratio
WHO: World Health organization

## References

[1] Organization WH. Haemoglobin concentrations for the diagnosis of anaemia and assessment of severity. World Health Organization; 2011.

[2] WHO C. Assessing the iron status of populations: report of a joint World Health Organization/Centers for Disease Control and Prevention technical consultation on the assessment of iron status at the population level. World Health Organization, Geneva, Switzerland 2007.

[3] Organization WH. Comprehensive implementation plan on maternal, infant and young child nutrition. World Health Organization; 2014. 10.3945/an.114.007781 PMC4288273 25593153

[4] Organization WH. Global nutrition targets 2025: Policy brief series. World Health Organization; 2014.

[5] Organization WH. The global prevalence of anaemia in 2011. Geneva: World Health Organization; 2015. 2017.

[6] Haas JD, Brownlie T IV. Iron deficiency and reduced work capacity: a critical review of the research to determine a causal relationship. The Journal of nutrition. 2001;131(2):676S–90S. 11160598 10.1093/jn/131.2.676S

[7] Horton S, Ross J. The economics of iron deficiency. Food policy. 2003;28(1):51–75.

[8] Brabin BJ, Hakimi M, Pelletier D. An analysis of anemia and pregnancy-related maternal mortality. The Journal of nutrition. 2001;131(2):604S–15S. 11160593 10.1093/jn/131.2.604S

[9] Mawani M, Ali SA, Bano G, Ali SA. Iron deficiency anemia among women of reproductive age, an important public health problem: Situation analysis. Reproductive System & Sexual Disorders: Current Research. 2016;5(3):1.

[10] World Health Organization. Global Nutrition Targets 2025: Anemia Policy Brief Geneva: WHO; 2014 [10 March 2019]. https://apps.who.int/iris/bitstream/handle/10665/148556/WHO_NMH_NHD_14.4_eng.pdf?ua=1.

[11] Terefe B, Birhanu A, Nigussie P, Tsegaye A. Effect of maternal iron deficiency anemia on the iron store of newborns in Ethiopia. Anemia. 2015;2015. 10.1155/2015/808204 PMC4334859 25734012

[12] Kumar KJ, Asha N, Murthy DS, Sujatha M, Manjunath V. Maternal anemia in various trimesters and its effect on newborn weight and maturity: an observational study. International journal of preventive medicine. 2013;4(2):193. 23543625 PMC3604852

[13] Dane B, Arslan N, Batmaz G, Dane C. Does maternal anemia affect the newborn. Özgün Araştırma. 2013:195–9.

[14] De Benoist B, Cogswell M, Egli I, McLean E. Worldwide prevalence of anaemia 1993–2005; WHO Global Database of anaemia. 2008. 10.1017/S1368980008002401 18498676

[15] Nojilana B, Norman R, Bradshaw D, Van Stuijvenberg ME, Dhansay MA, Labadarios D, et al Estimating the burden of disease attributable to vitamin A deficiency in South Africa in 2000. South African Medical Journal. 2007;97(8):748–53. 17952233

[16] Unicef U, WHO U. WHO: Iron deficiency anaemia: assessment, prevention, and control. A guide for programme managers. 2001.

[17] McLean E, Cogswell M, Egli I, Wojdyla D, De Benoist B. Worldwide prevalence of anaemia, WHO vitamin and mineral nutrition information system, 1993–2005. Public health nutrition. 2009;12(4):444–54. 18498676 10.1017/S1368980008002401

[18] Stevens GA, Finucane MM, De-Regil LM, Paciorek CJ, Flaxman SR, Branca F, et al Global, regional, and national trends in haemoglobin concentration and prevalence of total and severe anaemia in children and pregnant and non-pregnant women for 1995–2011: a systematic analysis of population-representative data. The Lancet Global Health. 2013;1(1):e16–e25. 25103581 10.1016/S2214-109X(13)70001-9 PMC4547326

[19] Kassebaum NJ. The global burden of anemia. Hematology/Oncology Clinics. 2016;30(2):247–308. 27040955 10.1016/j.hoc.2015.11.002

[20] Hakizimana D, Nisingizwe MP, Logan J, Wong R. Identifying risk factors of anemia among women of reproductive age in Rwanda–a cross-sectional study using secondary data from the Rwanda demographic and health survey 2014/2015. BMC Public Health. 2019;19(1):1662. 31829161 10.1186/s12889-019-8019-z PMC6907339

[21] Nankinga O, Aguta D. Determinants of Anemia among women in Uganda: further analysis of the Uganda demographic and health surveys. BMC Public Health. 2019;19(1):1–9. 31888579 10.1186/s12889-019-8114-1 PMC6937990

[22] Massawe SN, Urassa EN, Nyström L, Lindmark G. Anaemia in women of reproductive age in Dar-es-Salaam, Tanzania. East African medical journal. 2002;79(9):461–6. 12625686 10.4314/eamj.v79i9.9117

[23] World Bank. Zambia—Prevalence Of Anemia Among Women Of Reproductive Age (% Of Women Ages 15–49). 2016.

[24] World Bank. Burundi—Prevalence Of Anemia Among Women Of Reproductive Age (% Of Women Ages 15–49). 2016.

[25] Debelo O, Shiferaw Y. Correlates of Anemia Status Among Women of Reproductive Age in Ethiopia. SSRN 3463707. 2019.

[26] World Bank. Malawi—Prevalence Of Anemia Among Women Of Reproductive Age (% Of Women Ages 15–49). 2016.

[27] Bezerra AGN, Leal VS, Lira PICd, Oliveira JS, Costa EC, Menezes RCEd, et al Anemia and associated factors in women at reproductive age in a Brazilian Northeastern municipality. Revista Brasileira de Epidemiologia. 2018;21:e180001. 10.1590/1980-549720180001 30088588

[28] Kandala NI, Pallikadavath S, Channon AA, Knight G, Madise NJ. A multilevel approach to correlates of anaemia in women in the Democratic Republic of Congo: findings from a nationally representative survey. European Journal of Clinical Nutrition. 2020;74(5):720–31. 31685966 10.1038/s41430-019-0524-8

[29] Habyarimana F, Zewotir T, Ramroop S. Spatial Distribution and Analysis of Risk Factors Associated with Anemia Among Women of Reproductive Age: Case of 2014 Rwanda Demographic and Health Survey Data. The Open Public Health Journal. 2018;11(1).

[30] Kibret KT, Chojenta C, D’Arcy E, Loxton D. Spatial distribution and determinant factors of anaemia among women of reproductive age in Ethiopia: a multilevel and spatial analysis. BMJ open. 2019;9(4):e027276. 10.1136/bmjopen-2018-027276 PMC6500301 30948614

[31] Lover AA, Hartman M, Chia KS, Heymann DL. Demographic and spatial predictors of anemia in women of reproductive age in Timor-Leste: implications for health program prioritization. PloS one. 2014;9(3). 10.1371/journal.pone.0091252 PMC3954687 24632676

[32] Liyew AM, Teshale AB. Individual and community level factors associated with anemia among lactating mothers in Ethiopia using data from Ethiopian demographic and health survey, 2016; a multilevel analysis. BMC Public Health. 2020;20(1):1–11. 32448212 10.1186/s12889-020-08934-9 PMC7247135

[33] Lakew Y, Biadgilign S, Haile D. Anaemia prevalence and associated factors among lactating mothers in Ethiopia: evidence from the 2005 and 2011 demographic and health surveys. BMJ open. 2015;5(4):e006001. 10.1136/bmjopen-2014-006001 PMC4401847 25872935

[34] Gautam S, Min H, Kim H, Jeong H-S. Determining factors for the prevalence of anemia in women of reproductive age in Nepal: Evidence from recent national survey data. PloS one. 2019;14(6). 10.1371/journal.pone.0218288 PMC6561639 31188883

[35] Win HH, Ko MK. Geographical disparities and determinants of anaemia among women of reproductive age in Myanmar: analysis of the 2015–2016 Myanmar Demographic and Health Survey. WHO South-East Asia journal of public health. 2018;7(2):107–13. 30136669 10.4103/2224-3151.239422

[36] Sadeghian M, Fatourechi A, Lesanpezeshki M, Ahmadnezhad E. Prevalence of anemia and correlated factors in the reproductive age women in rural areas of tabas. Journal of family & reproductive health. 2013;7(3):139. 24971116 PMC4064781

[37] Ali JH. Gender differences in household headship and level of awareness on anaemia among Ethiopian women: Evidences from a nationwide cross-sectional survey. Ethiopian Journal of Health Development. 2018;32(2).

[38] Nisar R, Anwar S, Nisar S. Food security as determinant of anemia at household level in Nepal. Journal of Food Security. 2013;1(2):27–9.

[39] Alemu T, Umeta M. Reproductive and Obstetric Factors Are Key Predictors of Maternal Anemia during Pregnancy in Ethiopia: Evidence from Demographic and Health Survey (2011). Anemia. 2015;2015:649815. 26417454 10.1155/2015/649815 PMC4568321

[40] Klemm R, Sommerfelt AE, Boyo A, Barba C, Kotecha P, Steffen M, et al. Are we making progress on reducing anemia in women. Cross-country Comparison of Anemia Prevalence, Reach, and Use of Antenatal Care and Anemia Reduction Interventions. Washington DC, USA: A2Z: The USAID Micronutriend and Child Blindness Project. 2011 Jun.

[41] Mbule M, Byaruhanga Y, Kabahenda M, Lubowa A. Determinants of anemia among pregnant women in rural Uganda. Rural and remote health. 2012;13:2259. 23679828

[42] Kordas K, Centeno ZYF, Pachón H, Soto AZJ. Being overweight or obese is associated with lower prevalence of anemia among Colombian women of reproductive age. The Journal of nutrition. 2013;143(2):175–81. 23236023 10.3945/jn.112.167767

[43] Wiebe ER, Trouton KJ, Eftekhari A. Anemia in early pregnancy among Canadian women presenting for abortion. International Journal of Gynecology & Obstetrics. 2006 7;94(1):60–1. 16678824 10.1016/j.ijgo.2006.03.018

[44] Uche-Nwachi EO, Odekunle A, Jacinto S, Burnett M, Clapperton M, David Y, et al Anaemia in pregnancy: associations with parity, abortions and child spacing in primary healthcare clinic attendees in Trinidad and Tobago. African health sciences. 2010 3;10(1):66. 20811527 PMC2895803

[45] Arabyat RM, Arabyat G, Al-Taani G. Prevalence and risk factors of anaemia among ever-married women in Jordan. East Mediterr Health J. 2018;24(8). 10.26719/emhj.18.074 31612968

[46] Obse N, Mossie A, Gobena T. Magnitude of anemia and associated risk factors among pregnant women attending antenatal care in Shalla Woreda, West Arsi Zone, Oromia Region, Ethiopia. Ethiopian journal of health sciences. 2013;23(2):165–73. 23950633 PMC3742894

[47] Bekele A, Tilahun M, Mekuria A. Prevalence of anemia and its associated factors among pregnant women attending antenatal care in health institutions of Arba Minch Town, Gamo Gofa Zone, Ethiopia: a cross-sectional study. Anemia. 2016;2016. 10.1155/2016/1073192 PMC4779815 27022481

[48] Gebremedhin S, Asefa A. Association between type of contraceptive use and haemoglobin status among women of reproductive age in 24 sub-Saharan Africa countries. BMJ sexual & reproductive health. 2019;45(1):54–60. 10.1136/bmjsrh-2018-200178 30463847

[49] Adamu AL, Crampin A, Kayuni N, Amberbir A, Koole O, Phiri A, et al Prevalence and risk factors for anemia severity and type in Malawian men and women: urban and rural differences. Population health metrics. 2017;15(1):12. 28356159 10.1186/s12963-017-0128-2 PMC5371260

[50] Organization WH. Worldwide prevalence of anaemia 1993–2005: WHO global database on anaemia. 2008.

[51] WHO. Global Nutrition Targets 2025. AnaemiaPolicy Brief, Geneva. World Health Organization. 2014.

[52] DHS Methodology. Obtained from http://dhsprogram.com/What-We-Do/Survey-Types/DHS-Methodology.cfm. Accessed on 19 June 2017: DHS Program; [cited 2017 19 June 2017].

[53] Raghuram V, Anil M, Jayaram S. Prevalence of anaemia amongst women in the reproductive age group in a rural area in south india. Int J Biol Med Res. 2012;3(2):1482–4.

[54] Harding KL, Aguayo VM, Namirembe G, Webb P. Determinants of anemia among women and children in Nepal and Pakistan: An analysis of recent national survey data. Maternal & child nutrition. 2018;14:e12478. 28857410 10.1111/mcn.12478 PMC6586025

[55] Jamnok J, Sanchaisuriya K, Sanchaisuriya P, Fucharoen G, Fucharoen S, Ahmed F. Factors associated with anaemia and iron deficiency among women of reproductive age in Northeast Thailand: a cross-sectional study. BMC Public Health. 2020;20(1):102. 31992253 10.1186/s12889-020-8248-1 PMC6986100

[56] Saydam BK, Genc RE, Sarac F, Turfan EC. Prevalence of anemia and related factors among women in Turkey. Pakistan journal of medical sciences. 2017;33(2):433. 28523051 10.12669/pjms.332.11771 PMC5432718

[57] Little M, Zivot C, Humphries S, Dodd W, Patel K, Dewey C. Burden and determinants of anemia in a rural population in south India: A Cross-Sectional Study. Anemia. 2018;2018. 10.1155/2018/7123976 PMC6077670 30112198

[58] Trinh LTT, Dibley M. Anaemia in pregnant, postpartum and non pregnant women in Lak district, Daklak province of Vietnam. Asia Pacific journal of clinical nutrition. 2007;16(2). 17468088

[59] Soofi S, Khan GN, Sadiq K, Ariff S, Habib A, Kureishy S, et al Prevalence and possible factors associated with anaemia, and vitamin B 12 and folate deficiencies in women of reproductive age in Pakistan: analysis of national-level secondary survey data. BMJ Open. 2017;7(12):e018007. 10.1136/bmjopen-2017-018007 PMC5770950 29275342

[60] Nankinga, Olivia, Danstan Aguta, and Catherine Kabahuma. 2019. Trends and Determinants of Anemia in Uganda: Further Analysis of the Demographic and Health Surveys. DHS Working Paper No. 149. Rockville, Maryland, USA: ICF. 10.1186/s12889-019-8114-1 PMC6937990 31888579

[61] Arpey NC, Gaglioti AH, Rosenbaum ME. How socioeconomic status affects patient perceptions of health care: a qualitative study. Journal of primary care & community health. 2017;8(3):169–75. 10.1177/2150131917697439 PMC5932696 28606031

[62] Apouey BH. Health policies and the relationships between socioeconomic status, access to health care, and health. Israel journal of health policy research. 2013;2(1):50. 24354849 10.1186/2045-4015-2-50 PMC3878226

[63] Breymann C. Iron deficiency anemia in pregnancy. InSeminars in hematology 2015 Oct 1 (Vol. 52, No. 4, pp. 339–347). WB Saunders. 10.1053/j.seminhematol.2015.07.003 26404445

[64] Miller L, Hughes JP. Continuous combination oral contraceptive pills to eliminate withdrawal bleeding: a randomized trial. Obstetrics & Gynecology. 2003 4 1;101(4):653–61. 12681866 10.1016/s0029-7844(03)00014-0

[65] Glasier AF, Smith KB, Van der Spuy ZM, Ho PC, Cheng L, Dada K, et al Amenorrhea associated with contraception—an international study on acceptability. Contraception. 2003 1 1;67(1):1–8. 12521650 10.1016/s0010-7824(02)00474-2

