# Supplementary material for: Anemia and its associated factors among women of reproductive age in eastern Africa: A multilevel mixed-effects generalized linear model
Source: PLoS One. 2020 Sep 11;15(9):e0238957. doi: 10.1371/journal.pone.0238957 (PMC7485848; doi:10.1371/journal.pone.0238957)
Supplement: Attachment — Submitted filename: Response to reviewer.docx [file pone.0238957.s001.docx]

**Date: August 10, 2020**

**Author’s response to editor and reviewer**

**Title:** Prevalence and associated factors of anemia among reproductive-aged women in Eastern Africa; a multilevel analysis of the recent DHS data.

**Manuscript number**: PONE-D-20-18978

**Dear editor/reviewer:** We have now thoroughly updated the manuscript. We hope the improvements are sufficient and if that is not the case, any suggestions/comments are welcome. Below we present a point-by - point response to the questions raised by the editor & reviewer, to whom we thank for their valuable contributions to this research.

**Editor’s comment**

1. Please ensure that your manuscript meets PLOS ONE's style requirements, including those for file naming.

**Author’s response:** Dear editor thank you. We amended our manuscript according to the journal style.

**Reviewer #1: Dr. Paddy Ssentongo**
1. Change “Anemia in reproductive age women” to “Anemia in women of reproductive age."

**Author’s response:** thank you for the comment we amended to read as “anemia in reproductive age women” throughout the revised document (see the track change)
2. For culture appropriateness, “restrain to “low- and middle-income countries" as opposed to "developing countries."

**Author’s response:** Corrected in the revised paper.
3. Start a new sentence with “This study aimed to”

**Author’s response:** Amended in the revised paper
4. Change” …using the nine eastern African countries DHS data” to using DHS data of 9 eastern African countries,”

**Author’s response:** Corrected in the revised manuscript
5. Defined DHS at the first mention or avoid abbreviation in the abstract.

**Author’s response:** We first mentioned it as “Demographic and Health Survey (DHS)” in the revised manuscript
6. Delete “and variables with p-value <0.05 in the multivariable analysis were declared as significant determinants of anemia."

**Author’s response:** Thank you. We deleted in the revised manuscript.
7. Line 50: delete "developing."

**Author’s response:** Deleted
8. Line 50-57: For coherence, separate the effect of anemia women and their offspring separately (paragraph 2 line 53-58).

**Author’s response:** Thank you for the important concern you raised. We consider it and put the effects of anemia for women and the newborn/offspring separately.
9. Line 63-64: Add in 2011. As you know, the prevalence should be stated with a time period.

**Author’s response:** The time (2011) is added in the revised manuscript.
10. Remove "developing countries" to replace them with LMIC.

**Author’s response:** Thank you we removed it
11. Please clearly state the objective of threw study in the last paragraph of the background before starting line 80.

**Author’s response: thank you.** We consider it in the revised manuscript (see the last paragraph of the background section).
12. In addition, please state your hypothesis

**Author’s response:** We stated the hypothesis in the revised manuscript (found in the last paragraph of the revised manuscript).
13. Change "method" to "methods."

**Author’s response:** Changed
14. Cite line 90

**Author’s response:** Thank you. We add citation/reference
15. Please write all “reproductive-age women" to "women of reproductive age."

**Author’s response:** We amended it throughout the revised manuscript
16. Give the rationales for choosing the confounding factors to adjust for.

**Author’s response:** Thank you for raising the important concern. We choose the confounding factors based on reviewing of literatures, based on clinical judgment/importance, as well as based on their availability in the DHSs.
17. Line 115: The DHS uses a different categorization of wealth index, please use them as opposed to poorest/poorer

**Author’s response:** Thank you for your comment. We amended the terms to read as household wealth quantiles [first, second, middle, fourth, and highest] in the revised manuscript.
18. Line 124: Since the prevalence of the outcome of interest is very high (35%), the odds ratio can overestimate the prevalence ratio using logistic regression. Although the use of odds ratios is correct, authors should consider prevalence ratios analysis by employing either log-binomial or Poisson regression “see Barros, A. J., & Hirakata, V. N. (2003). Alternatives for logistic regression in cross-sectional studies: an empirical comparison of models that directly estimate the prevalence ratio. BMC medical research methodology, 3(1), 21. See use of log-binomial analysis in anemia prevalence" <https://link.springer.com/article/10.1186/s12884-020-03064-x>]

**Author’s response:** We really thank you for this important concern and direction. We were trying to fit a multilevel log-binomial model in order to calculate the prevalence ratio (rather than the odds ratio) for a clustered binary outcome using stata [using the command *meglm depvar indvar || v001: , family(binomial) link(log) eform* ], unfortunately we have got an unexpected error which says *link log is not allowed with family Bernoulli*. Therefore, we consider a reasonable analytic option that is we use Poisson regression with robust standard errors to overcome the over estimation of the prevalence ratio (an important measure of association if the outcome of interest is common) as stated by [**Coutinho L, Scazufca M, Menezes PR, 2008, Zou G, 2004, and Barros, A.J., Hirakata, V.N, 2003**]**.**

19. Describe the methods of sample weighting. Was additional weighting with the population of the country done?

**Author’s response:** Just we appended the data and we weight it using the weighting factor.
20. Line 134: Authors should explain why started methods of model comparison such as AIC/BIC were not used
**Author’s response:** Since the models were nested we used deviance (the preferable method for model comparison for nested models)
21. I recommend adding a geographical map depicting the distribution of anemia in East Africa.
**Author’s response:** Thank you for your comment. We added the map displaying the prevalence of anemia in the revised manuscript (see figure 2).
22. As stated on line 247-248, infectious diseases such as malaria and HIV substantially contribute to anemia in the population in LMICs. However, the authors never explored their effect in this study, and yet malaria and HIV data are available in the DHS records. For the recent similar study for the west and central Africa, see Ssentongo, P., Ba, D. M., Ssentongo, A. E., Ericson, J. E., Wang, M., Liao, D., & Chinchilli, V. M. (2020). Associations of malaria, HIV, and coinfection, with anemia in pregnancy in sub-Saharan Africa: a population-based cross-sectional study. BMC Pregnancy and Childbirth, 20(1), 1-11.

**Author’s response:** Thank you for your important question. Even though infectious diseases (such as malaria, HIV/AIDS, and intestinal parasites) are known to contribute to anemia, we used the IR (individual women) data set, for this study, in which there was no such variables in most of the surveys. Moreover, they are found as separate file/data and difficult to access and append these data. For example, in Ethiopia information about malaria is not found in the EDHS 2016 (IR data set), rather it is found as separate data (as malaria indicator survey). Therefore, we acknowledge it as limitation of our study (see the limitation section)
23. The should consider cut down the discussion to focus on the main findings and continue to provide 2-3 paragraphs explaining their results in light of other studies. The final section should be the clinical and public health implications and finally, the limitations. The authors do not have to discuss every result.

**Author’s response:** Dear reviewer thank you for your important concern. Even though. it is difficult to cut down the discussion section in to 2/3 paragraphs (since we had many findings) we tried to concentrate and discuss the most important variables. We also putted the strength/implications, as well as the limitations of our study in the last paragraph of the discussion section.

**Dear reviewer**

We made a re-analysis based on your concern / comment, as well as due to the inclusion of Zambia DHS (2018), which was previously missed in view of the previous Zambian DHS (ZDHS 2013). So, our study was based on 10 East African DHSs with a weighted sample of 101524 women of reproductive age.

**References**

*Coutinho L, Scazufca M, Menezes PR.* ***Methods for estimating prevalence ratios in cross-sectional studies.*** *Revista de saude publica. 2008 Dec;42(6):992-8.*

*Zou G. A modified poisson regression approach to prospective studies with binary data. American journal of epidemiology. 2004 Apr 1;159(7):702-6.*

*Barros, A.J., Hirakata, V.N.* ***Alternatives for logistic regression in cross-sectional studies: an empirical comparison of models that directly estimate the prevalence ratio****. BMC Med Res Methodol* ***3,*** *21 (2003).* [*https://doi.org/10.1186/1471-2288-3-21*](https://doi.org/10.1186/1471-2288-3-21)

*Blumenberg, Cauane. (2017).* ***Re: How can we calculate prevalence ratio from Poisson regression****?. Retrieved from: https://www.researchgate.net/post/How_can_we_calculate_prevalence_ratio_from_Poisson_regression/58744263615e27dd6f318ca7/citation/download.*
